# External Use of Cod Liver Oil

**Published:** 1852-09

**Authors:** 


*External Use of Cod Liver Oil.*—Dr. A. H. David recommends (*Canada Medical Journal,* May, 1852) the cod-liver oil as a local application in various cutaneous affections, and states that after a trial of it in such cases for upward of two years, he has found it to act almost specifically.

In ringworm of the scalp, Dr. D. says he has used it in more than twenty cases with entire success. Some cases, which had resisted other methods of treatment for weeks, were cured in four or five days.

He has also used it in tinea capitis with equal success; and he cured one case of psoriasis inveterata of three years standing, by this application, in seven weeks.—*American Jour. of Med. Sciences.*

